# Breast Cancer Metastasis to the Pituitary Presenting With Apoplexy

**DOI:** 10.7759/cureus.94287

**Published:** 2025-10-10

**Authors:** Badar Ahmad, Tajuddin H Mohammed, Palani Nagappan, Haissan Iftikhar, Shahz Ahmed

**Affiliations:** 1 General Surgery, East Surrey Hospital, Surrey and Sussex NHS Trust, Redhill, GBR; 2 Internal Medicine, East Surrey Hospital, Surrey and Sussex NHS Trust, Redhill, GBR; 3 Otolaryngology, University of Cambridge, Cambridge, GBR; 4 Otolaryngology - Head and Neck Surgery, Rawal Institute of Health Sciences, Islamabad, PAK; 5 ENT, Royal Wolverhampton NHS Trust, Wolverhampton, GBR

**Keywords:** bitemporal hemianopia, breast cancer metastases, pituitary apoplexy, suprasellar region, transsphenoidal endoscopic debulking

## Abstract

A 66-year-old female was referred to the skull base surgeons with diplopia, worsening bilateral visual disturbances, headaches, polydipsia, and polyuria. For the past 17 years, she had been treated for recurrent breast cancers and sigmoid colon carcinoma surgically and radiologically with aromatase inhibitors. MRI scans indicated significant metastases in her liver, bones, breast, pituitary, and left orbit with infiltration into the left optic nerve. During the investigations, the proximity of the large lesion to the carotid arteries and optic nerves had to be taken into account, while balancing the risk of stroke and further visual loss from surgery and her current worsening symptoms. Due to the terminal nature of the disease and atypical presentation of diplopia, a multidisciplinary discussion was conducted to decide the best course of treatment to provide symptomatic relief. Afterward, the surgical resection of the lesion was performed successfully with no complications.

This report highlights the fact that metastatic cancers can have unusual and atypical presentations of existing diseases, such as sixth nerve palsy seen in addition to the typical symptoms of pituitary adenoma. Furthermore, radiological evidence in identifying affected structures is also important in identifying the etiology of the lesion based on previous cases. Lastly, the report emphasizes the significance of multidisciplinary discussions in determining the most appropriate management plan to ensure symptomatic relief in terminal diseases.

## Introduction

Breast cancer is the most frequently diagnosed malignancy among women and a leading cause of cancer-related mortality worldwide. Metastatic disease commonly involves the bone, lung, liver, and brain, while spread to the pituitary gland is uncommon, representing less than 1% of all pituitary tumors [1,2,3]. In autopsy series, pituitary involvement has been documented in 6-29% of patients with advanced breast cancer, though most remain asymptomatic during life [4,5]. Metastases typically affect the posterior pituitary, likely due to its direct arterial blood supply, whereas anterior pituitary involvement usually results from extension of disease from surrounding structures [6,7]. Clinical features can resemble those of pituitary adenomas, including headache, visual field loss, ophthalmoplegia, and hypopituitarism. Diabetes insipidus is considered more suggestive of metastasis than adenoma [2]. On imaging, pituitary metastases are difficult to distinguish from benign adenomas, and histopathology is often required for definitive diagnosis [2,5].

Pituitary apoplexy, defined as acute hemorrhage or infarction of the pituitary gland, is a neurosurgical emergency most often associated with macroadenomas. Its occurrence secondary to metastatic disease is exceptionally rare, with only a small number of reported cases [2]. We describe the case of a 66-year-old female with a history of recurrent breast cancer and sigmoid colon carcinoma who presented with acute visual compromise due to pituitary metastasis complicated by apoplexy. This report highlights the diagnostic challenges, the role of imaging and multidisciplinary decision-making, and the importance of early intervention to preserve vision and provide symptomatic relief in advanced disease.

## Case presentation

A 66-year-old female with a history of breast and colon cancer presented with diplopia and worsening bilateral visual disturbances, more prominent on the right side. She also had headaches, polydipsia and polyuria. Previously, she had been diagnosed and treated for left-sided breast cancer with curative intent, including surgery and letrozole with adjuvant radiotherapy approximately 10 years before her most recent visit. During a follow-up, it had been noted that she had developed metastases to the liver, left orbit, bones, right breast and pituitary gland. Initially, the large suprasellar pituitary metastases had been presumed to be a pituitary macroadenoma.

**Figure 1:**
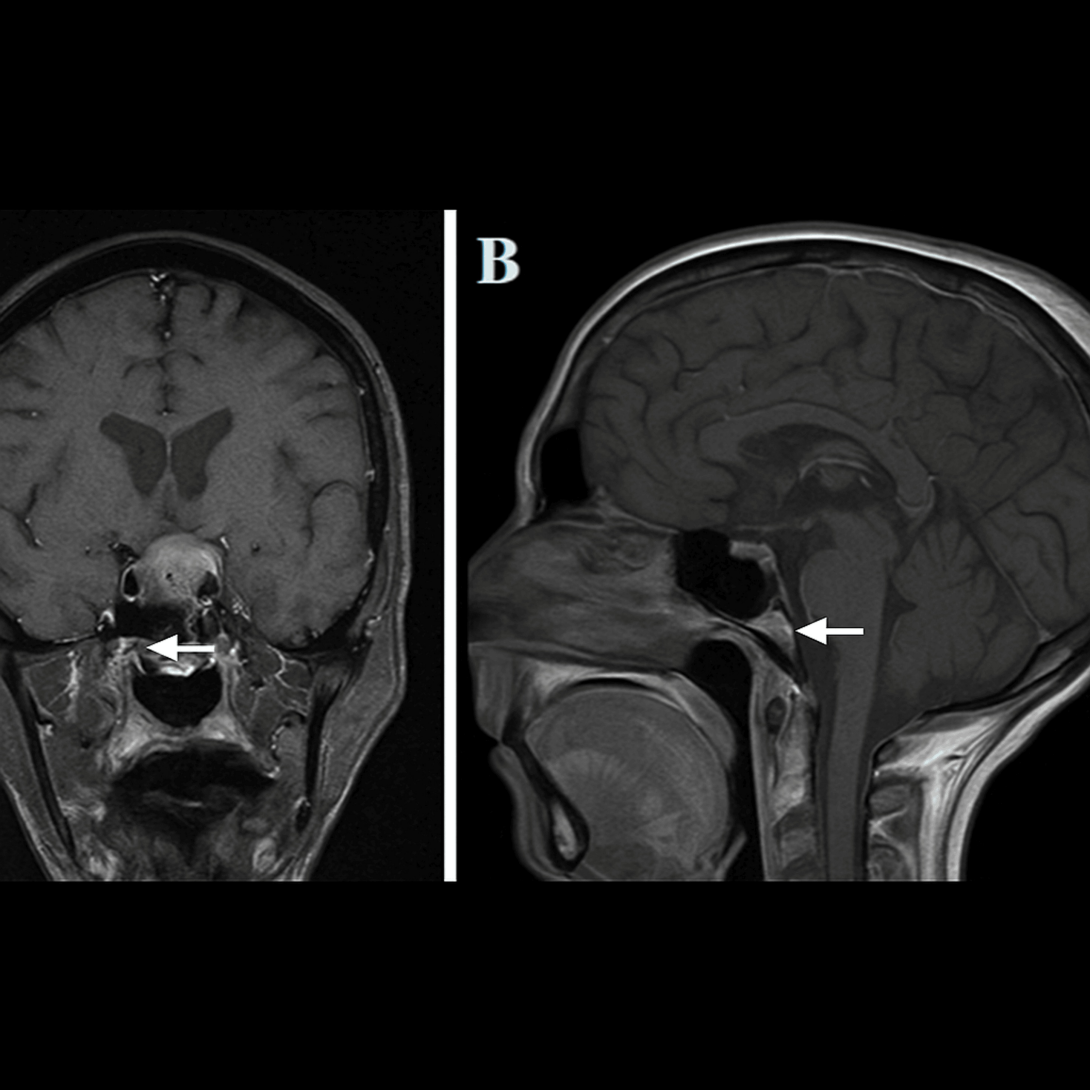
MRI pituitary post-contrast revealed a heterogeneously enhancing pituitary lesion extending into the suprasellar compartment (A) Preoperative T1 MRI with contrast – coronal view. (B) Preoperative T1 MRI with contrast – sagittal view MRI: magnetic resonance imaging

Clinical examination revealed impaired abduction of the left eye. Colour vision was intact, and there were signs of diplopia. MRI pituitary post-contrast revealed a heterogeneously enhancing pituitary lesion extending into the suprasellar compartment measuring 24 x 16 x 26 mm with significant superior displacement and compression of the optic chiasm. The lesion extended laterally to the right, causing partial encasement of the right internal carotid artery with no evidence of significant compression. There was a patchy loss of fat signal in the adjacent clivus with thinning and minimal expansion of the sella turcica, along with the previously seen skull osseous metastasis. The enhancing soft tissue disease seen in the left retro-bulbar region corresponded to a T2 iso to hypointense, avidly enhancing left retro-bulbar soft tissue mass mainly confined to the intraconal compartment with infiltration of the intraocular and intra-orbital parts of the left optic nerve (Figure 1).

No significant change in size was appreciated when compared to the previous scan. Following discussion at the skull base multidisciplinary meeting, a decision was made to proceed with a transsphenoidal endoscopic debulking of the lesion due to imminent risk of irreversible vision loss, as the presumed cause of the visual disturbance was secondary to the mass effect of the lesion on the optic chiasm. The turbinates were lateralized, and the sphenoid ostium was identified. The sphenoethmoidal recess was decongested, and bilateral rescue flaps were raised using the Colorado needle. Following this, a posterior septectomy was done, and bilateral sphenoidotomy was also performed to remove the rostrum. A sphenoid septation was drilled using a 4.3 mm diamond burr with a sellotomy to the “Four blues”, which are landmarks comprising the cavernous sinuses laterally, the superior and inferior intercavernous sinus rostrocaudally. Following the removal of the bone over the sella, a cruciate incision was made in the pituitary dura to excise the tumour, where fragments of the yellow tumour came out en bloc. This tissue was not seen to be invading the surrounding structures.

**Figure 2:**
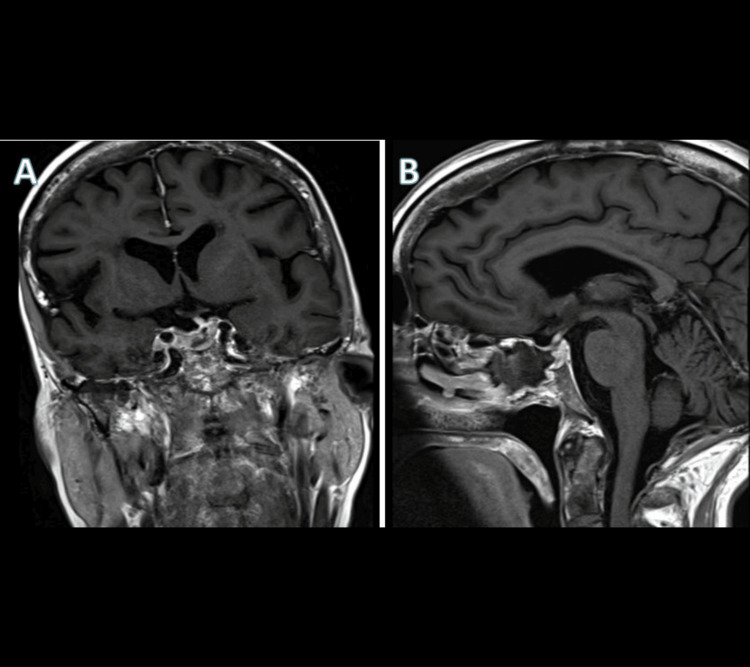
Postoperative T1 MRI with contrast (A) Coronal view. (B) Sagittal view MRI: magnetic resonance imaging

The procedure was uneventful. Histology of the biopsy confirmed the diagnosis as metastatic cancer, consistent with pleomorphic lobular carcinoma of breast origin. HER2/CEP17 ratio was 1.07, below the threshold to be considered HER2-positive. There was no HER2 gene amplification with a negative oestrogen receptor (ER), Progesterone receptor (PR) and HER2 (triple negative). Postoperatively, the patient had improvement in her visual fields, and a follow-up MRI demonstrated a gross removal of the lesion (Figure 2). Following the operation, the patient had complete resolution of visual symptoms. An MRI scan was performed six months after the procedure, which showed no recurrence, and there were no pathological findings on clinical examination.

## Discussion

Metastases to the pituitary from any origin are exceedingly rare, making up less than 1% of pituitary tumours [2]. Typical symptoms seen in previous case reports on breast cancer metastases are similar to those of pituitary macroadenoma seen such as diabetes insipidus, visual field defects, headache, ophthalmoplegia, and anterior pituitary dysfunction [2]. However, this is the first case, to our knowledge, where this neoplasm resulted in pituitary apoplexy. Pituitary metastases were found to have occurred in 6-29% of breast cancer patients based on an autopsy series where histopathological examinations following palliative hypophysectomy in end-stage breast cancer [3-6].

HER2-positive or triple-negative breast cancers are known to metastasize mostly to the brain [5]. Neoplastic cells metastasize to the pituitary commonly through the direct blood supply to the posterior pituitary gland or seeding of these malignant cells by direct invasion through the skull base [5,7]. Diabetes insipidus occurs due to impaired functioning of the posterior pituitary gland, due to compression or invasion, thereby resulting in decreased secretion of anti-diuretic hormone, with other symptoms being headaches, anterior pituitary dysfunction, visual disturbances secondary to compression of the optic chiasm, and extraocular nerve palsies [2]. Our patient also had a cranial nerve six palsy in addition to the acute visual field loss.

Radiological findings are not sensitive in differentiating between benign and malignant lesions. On T1-weighted MRI, images of the posterior hypophysis show a reduction of high signal intensity or enlargement of the pituitary stalk, indicating metastases [5,6]. In our case, an iso- to hypo-intense avidly enhancing mass of left retrobulbar soft tissue on a T1-weighted scan was seen (Figure 1). This is primarily contained in the intraconal compartment with infiltration of the intraocular and intraorbital portions of the left optic nerve. Additionally, the opacity in the fat plane between the lesion and the medial rectus muscle may indicate invasion. Aligning with a meta-analysis by Komnios et al. [2] and the radiological findings reported specifically about these metastatic lesions, our case matches the radiological findings stated in the analysis and is thereby indicative of a metastatic pituitary lesion. Classically, metastatic pituitary lesions have a dumbbell-shaped intrasellar and suprasellar mass that extends laterally and superiorly into surrounding structures. This was seen in our case, where the tumour extended laterally into the region of the carotid arteries and superiorly into the optic chiasm, leading to visual disturbances.

## Conclusions

For clinicians involved in medical or surgical practice, it would be prudent to be aware of rare instances of patients presenting with acute visual disturbance against a background of metastatic cancer. Pituitary apoplexy secondary to metastatic cancer is a rare entity as described in this case report. Thorough clinical examination and diagnostic tools such as MRI are crucial for an optimal management plan. This report emphasises the importance of considering rare acute neuro-ophthalmic symptoms associated with triple-negative breast cancers. The role of imaging remains a key diagnostic tool in diagnosing these entities and prompting acute surgical intervention for symptom control and preventing complications.
